# Disseminated *emergomyces orientalis* infection in a patient with systemic lupus erythematosus

**DOI:** 10.3389/fcimb.2024.1401463

**Published:** 2024-09-02

**Authors:** Jun Luo, Na An, Yarong Liu, Yisheng Li

**Affiliations:** Mianyang Central Hospital, School of Medicine, University of Electronic Science and Technology of China, Mianyang, China

**Keywords:** emergomyces, emergomycosis, emergomyces orientalis, itraconazole, amphotericin B, mNGS (metagenomic next-generation sequencing)

## Abstract

A case of Eimonosis orientalis was reported in a 52-year-old male farmer who presented with cough, phlegm, fever, headache, and nausea for more than 4 days. Haemophilic cells and fungal spores were identified in the bone marrow smear and confirmed as Aemon orientalis by culture. The same bacteria were also isolated from blood cultures

## Introduction

Emergomycosis is a systemic fungal disease caused by the dimorphic pathogen *Emergomyces*, predominantly affecting immunocompromised individuals. This pathogen is a soil saprobe that typically exists as mycelium in the environment and transforms into either thick-walled, sterile adiaspores or small, round, thin-walled, yeast-like cells within host tissues. Currently, seven species with distinct geographic distributions have been identified (Vinayagamoorthy et al., 2023): *E. africanus*, *E. canadensis*, *E. crescens*, *E. europaeus*, *E. orientalis*, *E. pasteurianus*, and *E. sola*. Of these, *E. africanus* is the primary causative agent, followed by *E. pasteurianus* and *E. canadensis*. Humans can contract the infection by inhaling contaminated spores, which then convert to yeast-like cells *in vivo*. These cells can cause extrapulmonary disease through hematological spread in susceptible hosts (Samaddar and Sharma, 2021a). The prevalence of infections caused by *Emergomyces* species is increasing worldwide. To date, only two cases of *E. orientalis* infection have been reported in China, in 2017 and 2021 (Wang et al., 2017; He et al., 2021). We now report an additional case of *E. orientalis* infection. The patient was diagnosed with emergomycosis upon presenting to the rheumatology and immunology department of our hospital with symptoms of cough, sputum, and fever, alongside systemic lupus erythematosus.

## Case description

The 52-year-old male patient from Aba Prefecture, a long-time farmer, has been experiencing recurrent chest pain and edema for over two years. He was diagnosed with systemic lupus erythematosus and lupus nephritis, which are also associated with chronic renal insufficiency and Chronic viral hepatitis B. Since May 2021, he has been undergoing long-term treatment with oral prednisone (25 mg daily) and hydroxychloroquine (0.2 g daily), while continuously monitoring blood routine, liver function, kidney function, immunoglobulin levels, complement levels, and urine analysis. Approximately three months ago, the patient presented with a cough and sputum, which showed improvement after receiving anti-infection treatment. Four days prior to admission, he developed a new onset of cough and yellow-white sputum following a cold, accompanied by fever, recurrent chills, headache, nausea, and other discomforts, leading to his hospitalization. The patient had no history of diabetes or familial genetic disorders. His skin examination revealed no abnormalities other than reduced elasticity.

At initial workup, his HIV antibody test was negative, and T-cell subpopulation analysis showed immune deficiency (total T cell count 186 cells/µL, CD4+ T-lymphocyte count 51 cells/µL, CD8+ T-lymphocyte count 113 cells/µL). Other tests showed elevated whole blood C-reactive protein at 190.46 mg/L, leukocyte count at 12.13×10^9/L, hemoglobin at 153 g/L, platelets at 70×10^9/L, procalcitonin at 1.31 µg/L, total protein at 61.72 g/L, and albumin at 34.1 g/L. Renal function tests indicated renal failure (urea 15.83 mmol/L, creatinine 243.5 µmol/L, cystatin C 3.08 mg/L). Serologic fungal markers revealed an elevated Platelia Aspergillus galactomannan assay at 6.81 (reference range <0.5), and a positive 1,3-beta-D-glucan test at 127.18 pg/mL (reference range <60 pg/mL). Coagulation tests were normal except for an increased D-dimer level (5.73 mg/L). Urine culture showed no bacterial growth after 48 hours, and sputum culture showed no fungal growth after seven days. Bone marrow examination showed active hyperplasia, a normal granulocyte to erythrocyte ratio, and the presence of hemophagocytic cells and fungal spores.

**Figure 1:**
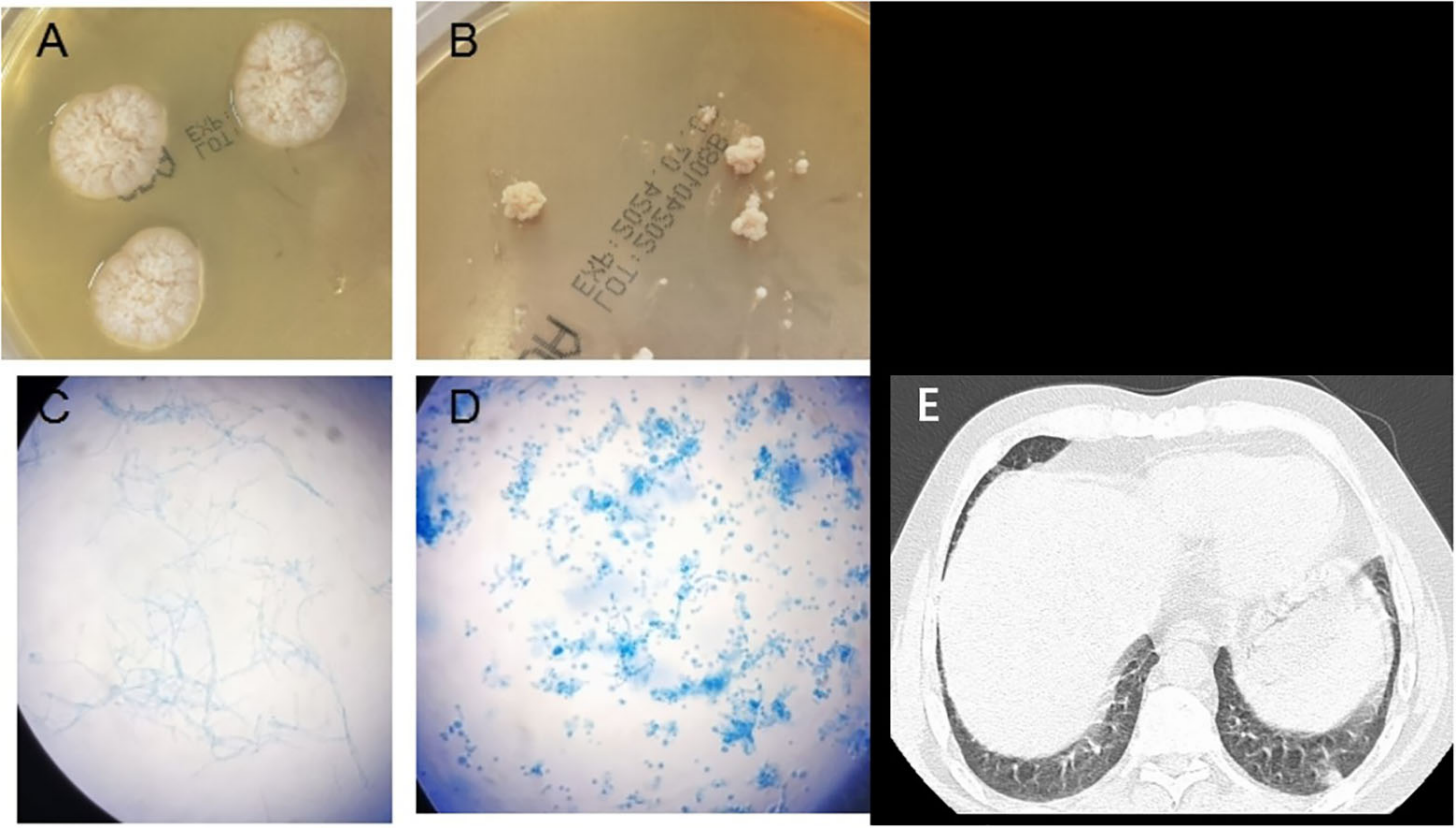
*Emergomyces orientalis* infection in a systemic lupus erythematosus patient. **(A)** Velvety white colonies of on Sabouraud agar on day 20 at 28°C. **(B)** White yeast-like colonies of on Sabouraud agar on day 20 at 35°C. **(C)** Colonies stained with lactophenol cotton blue showing hyphae and conidiophores (incubated at 28°C) (original magnification ×400). **(D)** Colonies stained with lactophenol cotton blue showing oval, budding forms consistent with small yeasts (incubated at 35°C) (original magnification ×400). **(E)** Computed tomography showed a small nodule in the right middle lobe of the lung.

Computed tomography (CT) of the chest showed a micronodule (**Figure 1**) in the middle lobe of the right lung, scattered interstitial changes in both lungs, a few localized areas of atelectasis, multiple enlargements of mediastinal and right cervical lymph nodes, and slight pleural effusions bilaterally.

Given the patient’s prolonged use of immunosuppressive drugs and positive G/GM tests, fluconazole was initiated to prevent fungal infection. Despite this, the patient continued to experience recurrent fevers, and infection markers increased. Consequently, piperacillin/tazobactam was added to the treatment regimen. On the sixth day of hospitalization, a bone marrow smear revealed hemophagocytic cells and yeast-like fungi, raising suspicions of *Histoplasma capsulatus* infection. Therefore, a repeat bone marrow puncture and mNGS were performed to identify the pathogen. Initial cultures of bone marrow and blood remained negative. Following the bone marrow smear results, the samples were removed from the blood culture system, centrifuged, and the precipitates inoculated on Sabouraud agar, cultured at both 28°C and 35°C. By the tenth day of admission, the mNGS report confirmed *Emergomyces orientalis* infection. After consulting with infectious disease and respiratory specialists, fungal septicemia was diagnosed. Treatment with intravenous amphotericin B cholesterol sulfate complex was initiated, starting at 50 mg per day and escalating to 150 mg within three days, in combination with piperacillin/tazobactam (4.5 g every 12 hours). Concurrently, methylprednisolone sodium succinate (40 mg daily) was administered intravenously due to the patient’s systemic lupus erythematosus. This regimen significantly reduced cough and sputum symptoms, and alleviated cold sensitivity, fever, abdominal pain, diarrhea, and other discomforts. On the twentieth day, a pathological examination of lymph nodes in the right supraclavicular area revealed numerous rounds, refractile bodies, consistent with a fungal infection. The effectiveness of the amphotericin B treatment prompted its continuation. After a month of therapy, the patient’s symptoms had significantly improved, leading to discharge with a diagnosis of disseminated emergomycosis. Post-discharge, itraconazole (0.2 g twice daily) was prescribed. Two months later, the patient remained stable.

**Figure 2:**
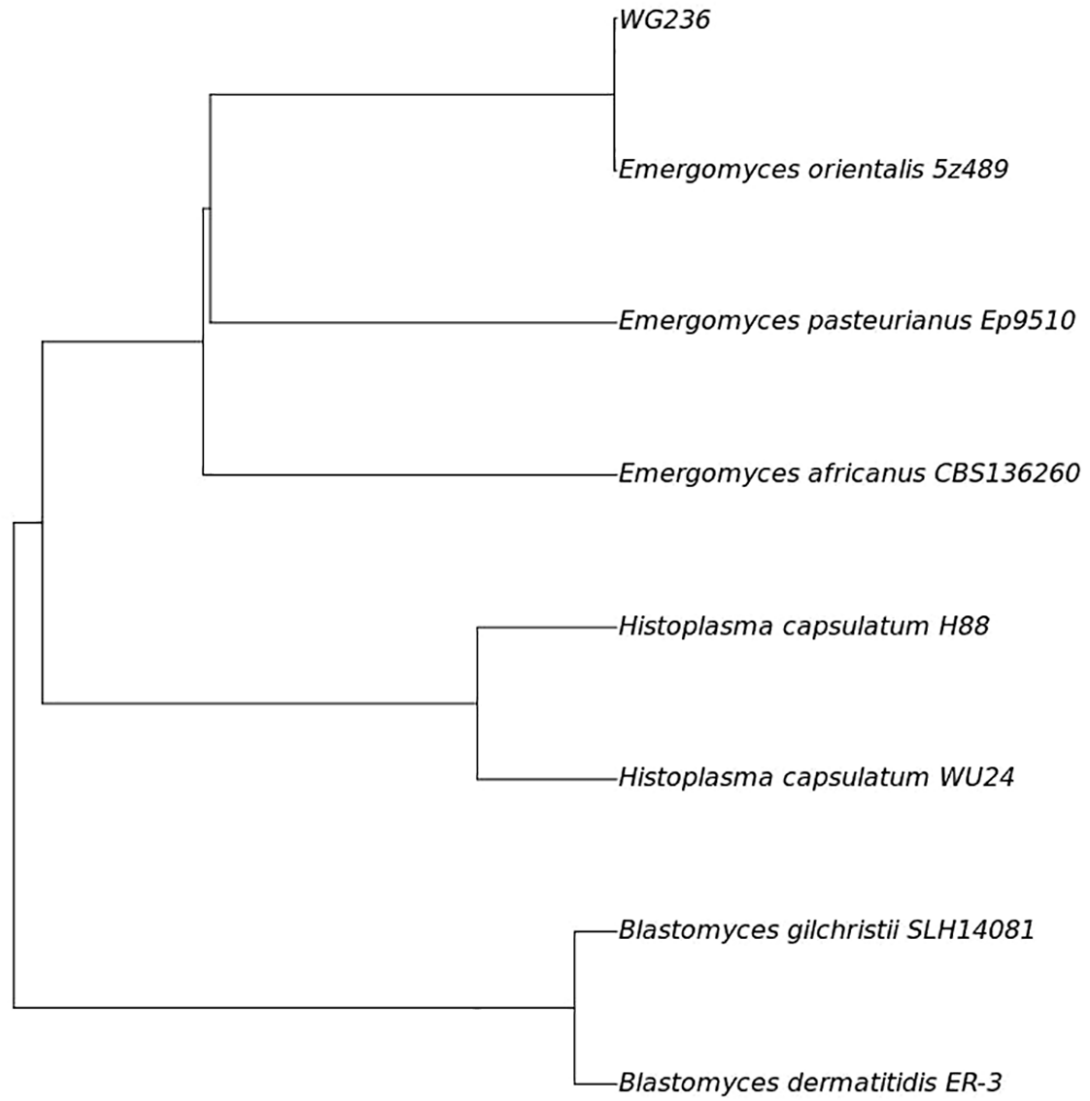
Fungal sequencing results (Phylogenomic tree based on the genome sequences of strains WG236 and the related members of the family Ajellomycetaceae.) ANI analysis showed that the ANI value of sample WG236 and Emergomyces orientalis was greater than 99.9%, indicating that they were identified as the same species.

Fungal colonies were observed in the patient’s bone marrow and blood cultures on the 15th day following centrifugation. The colonies incubated at 35°C appeared as white, yeast-like structures, while those grown on Sabouraud agar at 28°C exhibited mold characteristics (**Figure 1**). After 20 days of culture, a pure fungal colony (sample WG236) was sent to Changsha Jinyu Medical Inspection Office Co., Ltd. for sequencing. It was identified as *E. orientalis* (**Figure 2**).

## Discussion

*Emergomyces* is a newly recognized dimorphic fungus with reported cases across Asia, Europe, Africa, and the Americas. The majority of cases have been documented in South Africa (Mapengo et al., 2022; Ibe et al., 2023), where soil analyses revealed a 30% positivity rate for *E. africanus*, the primary causative agent of emergomycosis in the region (Schwartz et al., 2018). Research has identified significant risk factors for emergomycosis, including human immunodeficiency virus (HIV) infection, organ transplantation, malignancies, and the use of immunosuppressive drugs (Schwartz et al., 2019; Samaddar and Sharma, 2021b). It has also been reported (Schwartz et al., 2019) that individuals with ostensibly healthy immune function can contract this fungus. Clinical manifestations of emergomycosis typically include skin lesions, pulmonary infections, fungemia, and severe disseminated disease. The mortality rate among patients with emergomycosis ranges from 48 to 51% (Mapengo et al., 2022). Males are more frequently affected (Vinayagamoorthy et al., 2023), as evidenced by this case.

Current studies have identified seven species within the genus *Emergomyces*, including *E. orientalis*, of which only two cases have been reported in China. Similar to previous reports (Wang et al., 2017; He et al., 2021), immunodeficiency was the sole identified risk factor in these cases. It has been shown (Vinayagamoorthy et al., 2023) that emergomycosis predominantly affects HIV-infected patients, particularly those with a CD4 T lymphocyte count below 100 cells/μL. Among patients not infected with HIV, taking immunosuppressive drugs is a significant risk factor for emergomycosis. In such HIV-uninfected individuals, emergomycosis primarily presents as a respiratory disease, whereas in HIV-infected patients, the disease tends to disseminate, as evidenced by higher positive culture rates in blood and bone marrow specimens. In this case, although pulmonary inflammation was noted, no lung puncture was performed, thus it remains unclear whether *Emergomyces* was present in the lungs. Initially, cultures of blood and bone marrow did not yield any positive results. When fungal spores were detected in the bone marrow smear, the culture duration in the blood culture instrument was extended to 30 days, yet still showed no growth. Possible explanations for this include the slow growth rate of *Emergomyces* and insufficient CO2 production to trigger the alarm threshold of the culture instrument, which might also explain the low isolation rate of *Emergomyces* in blood and bone marrow of HIV-uninfected patients. Given the initial observation of yeast-like fungi in phagocytes on bone marrow smears, mNGS testing was conducted on the bone marrow, yielding positive results after re-centrifugation of samples from the initial blood culture. The lack of awareness about this pathogen can lead to misidentification as other fungal infections, such as *Histoplasma capsulatum* or *Penicillium marneffei*, which also show histiocytes with phagocytosed yeast cells. Therefore, this case highlights that detection of *Emergomyces* by traditional culture methods is not only time-consuming but also prone to misses, potentially delaying diagnosis and treatment.

The results of the fungal serologic markers, galactomannan and 1,3-β-D glucan, in this report were positive, which is contrary to previous reports (Gori et al., 1998; Pierce et al., 2023). It should be noted that due to the presence of cross antigen with histoplasmosis (Gori et al., 1998), these detection methods are not specific for emergomycosis. However, they can suggest clinicians remain vigilant against the risk of fungal infection and actively search for etiological evidence.

Emergomycosis is a systemic fungal disease with a high mortality rate, primarily prevalent in regions of Africa where AIDS is common. Recently, the disease has appeared in non-endemic areas and several new species have been identified. Due to the lack of specific clinical symptoms and limited diagnostic tools, clinical diagnosis of emergomycosis is challenging. Therefore, in patients with compromised cell-mediated immunity or those undergoing immunosuppressive therapy, fungal infection should be strongly suspected. Timely detection should employ molecular biological techniques, such as mNGS and PCR. Additionally, clinicians need to increase their awareness of emergomycosis to prevent misdiagnosis.

## Data Availability

The datasets presented in this study can be found in online repositories. The names of the repository/repositories and accession number(s) can be found in the article/supplementary material.
